# Design of Highly Sensitive NO_2_ Gas Sensors Based on Boron Nitride and Aluminum Nitride Two-Dimensional Materials: A DFT-Based Study

**DOI:** 10.3390/nano16171077

**Published:** 2026-08-29

**Authors:** Mohammed A. Al-Seady, Muaamar Hasan Idan, Ahmed Mesehour Ali Refaas, Mousumi Upadhyay Kahaly

**Affiliations:** 1 Department of Theoretical Physics, University of Szeged, Tisza Lajos Krt. 84-86, 6720 Szeged, Hungary; 2 Environmental Research and Studies Center, University of Babylon, Babylon 51001, Iraq; 3 Department of Physics, College of Science, University of Babylon, Babylon 51001, Iraq; sci.muaamar.hasan@uobabylon.edu.iq; 4 Ministry of Education, Al-Muthana 66001, Iraq; mybony19@gmail.com; 5 ELI-ALPS, ELI-HU Non-Profit Ltd., Wolfgang Sandner Utca 3, 6722 Szeged, Hungary

**Keywords:** boron nitride, aluminum nitride density functional theory, adsorption, two-dimensional materials

## Abstract

In the present study, the structural, electronic, optical, adsorption and sensitivity properties of BN and AlN nanoribbons towards nitrogen dioxide (NO_2_) gas molecules were investigated via density functional theory (DFT), DFT-D3 dispersion correction and time-dependent DFT (TD-DFT). Six different NO_2_ adsorption configurations were taken into account to evaluate the interaction between NO_2_ molecules and the BN and AlN nanoribbon surface. Three adsorption configurations were considered for each nanoribbon, resulting in six adsorption configurations in total. The adsorption energy calculations indicated stronger chemosorption on the AlN nanoribbon surface than the BN nanoribbon surface, while BN nanoribbons gave a stronger optical response than AlN. The charge transfer (CT) results conclude that the NO_2_ gas molecule acts like an electron donor, while it behaves like an electron acceptor on the AlN nanoribbon surface. The sensitivity (S) values confirm that the BN nanoribbon exhibits high sensing performance across all adsorption configurations, while the AlN nanoribbon shows the highest sensitivity for the H3 configuration. Furthermore, due to the chemisorption nature between BN and AlN nanoribbons’ surfaces, the band gap energy becomes narrower after interaction. For example, the band gap of the BN nanoribbons decreases from 6.2 eV to around 0.5 eV after NO_2_ adsorption, showing electron excitation and improving the electron sensing performance. Overall, the evaluated results indicate that AlN nanoribbons are promising candidates for adsorption-based NO_2_ gas sensors, while BN nanoribbons show superior potential for optical NO_2_ sensing applications.

## 1. Introduction

Day after day, environmental pollution increases due to rapid industrialisation. This directly affects the quality of breathable air due to the emission of many toxic gases like carbon monoxide (CO), nitrogen dioxide (NO_2_), and hydrogen di-sulfide (H_2_S) [1]. NO_2_ is considered one of the major pollutant gases that seriously affects all living beings. This is why researchers focus their attention toward reducing pollution in the environment via designing highly efficient gas-sensing devices [2,3]. One of the main research and development goals is the fabrication of novel materials for the gas-sensing field. In recent decades, graphene and its derivatives have become incresingly applied to various technologies, including gas sensors [4,5]. In 2004, the first graphene lattice was synthesized by Novoselov et al. at the Manchester Nanotechnology Laboratory [6]. These types of material have a flat surface structure; thus, all of the surface atoms interact with the environment directly. The most remarkable arrangement involving bonding of graphene and its derivatives is sp2 hybrid orbitals, which generate a tightly hexagonal honeycomb lattice [7]. Furthermore, they feature unique physical and chemical characteristics, like a high specific area, high carrier mobility, extreme mechanical properties, and acceptable thermal conductivity; thus, a wide range of applications is investigated, including ion batteries, solar cells, photonic devices and gas sensors [8]. Some of the most traditional graphene derivatives are II–VI and III–V compounds; group-III nitrides have remarkable properties such as a long-ranging surface area, high flexibility and stability [9,10]. The electronic and optical properties of boron nitride (BN) and aluminum nitride (AlN) have been investigated widely by researchers [11]. The band gap properties of BN and AlN hexagonal structures have been investigated by Beiranvand et al.; the optimum values of the band gap energy of BN and AlN are approximately 5.0 eV and 3.0 eV, respectively [12]. Monolayer and single-crystal BN and AlN were synthesized via chemical vapor deposition and vapor phase transport, respectively [13].

Nitrogen dioxide (NO_2_) gas sensing is of paramount importance in environmental monitoring and public health research [14,15], given its prevalence as a major air pollutant originating from combustion processes. Elevated NO_2_ levels, primarily stemming from vehicular emissions and industrial activities, pose substantial risks to respiratory health and are linked to cardiovascular diseases [16]. Accurate and sensitive NO_2_ detection is crucial for timely intervention and effective air quality management. Halide and oxide perovskites [17,18] have recently attracted significant attention due to their tunable electronic structure, high surface activity, and defect tolerance, which collectively enable enhanced gas adsorption and charge-transfer interactions essential for sensitive detection [19,20].

D. Mahdi et al., in (2019), investigated the adsorption energy between the surface of pure BN and carbon-doped BN. They deduced that the BN nanoribbon has good sensitivity for detecting NO_2_ gas molecules. Furthermore, the NO_2_ adsorption strength increased when BN was doped by carbon atoms. The inherent characteristics of nanoribbons derived from these materials demonstrate their high surface area, tunable electronic properties, chemical inertness, and low defect density. These collectively contribute to their exceptional suitability for this critical sensing task [21].

In 2013, Zargham B. Ali P. studied the adsorption energy of NO_2_ gas molecules on the surface of aluminum-nitride nanocones (AlNNC). They have investigated the effect of chemical adsorption on the electronic and charge transfer properties of the AlNNC structures. During the adsorption process, the energy gap shifted from 2.86 eV to 1.62 eV. In addition, the computed adsorption energy varied from −2.837 eV to −2.306 eV. The authors explained that the type of interaction between the AlNNC and NO_2_ gas molecules was chemical [22].

In 2020, Yogi R. and Jaiswal K. studied the suitability of III–V nitride nanoribbons for detecting NO_2_ gas molecules. They utilized boron nitride, aluminum nitride and gallium arsenide as NO_2_-based gas sensor materials. In addition, they used Quantum-ESPRESSO code for evaluating the electronic and adsorption properties. They computed the adsorption energy as a function of ribbon length. The maximum adsorption energy was −4.61 eV for the AlN nanostructures, higher than BN’s energy. The energy gap property of the BN and AlN nanoribbons shifted from 4.12 eV to 0.8 eV and from 2.05 eV to 3.1 eV, respectively [23].

In 2023, R. A. Taha et al. investigated the ability of pure and carbon-doped AlN nanocages to detect toxic gases such as SO_2_, NO_2_ and NH_3_ molecules. They utilized the DFT method for evaluating adsorption energies of the suggested nanocages. They found that the energy gap of pure AlN was about 4.1 eV, which reduced to 4.0 eV due to the effect of carbon atoms. The adsorption energy of NO_2_ gas on the surface of AlN was −0.19 eV. They clarified the presence of physical adsorption between AlN and the NO_2_ gas molecule. In addition, they improved the strength of gas sensing by doping AlN with carbon atoms. The adsorption energy rose to −3.65 due to impact of these carbon atoms. Finally, they clarified that the pure and carbon-doped AlN nanocages can be utilized as promising materials for removing toxic gases such as NO_2_ gas molecules [24].

T. A. Hussein et al. investigated the electronic and optical properties of BN bilayers as gas sensors for toxic gases via the DFT method. The simulation results show that the band gap value of the BN bilayer system is approximately 4.0 eV. The type of interaction between CO_2_ and NO_2_ gases is physisorption due to the lower charge transfer between the surface of BN and toxic gases. Overall, researchers claim that the BN-bilayer nanosystem cannot be utilized to detect NO_2_, SO_2_ and CO_2_ gas molecules [25].

The aim of the present study is to investigates the adsorption ability of NO_2_ molecules on the surface of BN and AlN nanoribbons. Three different types of adsorption configrations were taken into consideration for each nanoribbon to determine the most stable adsorption candidate. Then, the adsorption energy, charge transfer, electronic sensitivity and optical response of BN and AlN nanoribbons for NO_2_ gas molecule adsorption were systematically evaluated.

## 2. Methodology

In the present article, the structural, electronic and optical properties of BN and AlN nanoribbons are examined by density function theory (DFT) and time-dependent DFT (TD-DFT), and Gaussian 09 software is used to evaluate the quantum physics properties regarding BN and AlN systems. The 6-31G* basis set and B3LYP level of theory are utilized for describing the exchange correlation interaction term. The main reason for choosing the 6-31G*/B3LYP functional groups is due to their efficient accuracy in describing compact molecular systems built from carbon, nitrogen, oxygen, hydrogen, aluminum and silicon [26]. Furthermore, the Van der Waals dispersion correction (DFT-D3) was taken into consideration to enhance the reliability of the adsorption strength.

The relaxation structure of boron nitride (BN) and aluminum nitride (AlN) nanoribbons was generated utilizing nanotube modular software. BN consists of 34 boron and nitrogen atoms, whereas AlN consists of 34 aluminum and nitrogen atoms. The BN and AlN edges undergo hydrogenation in order to reduce the boundary effects.

The adsorption energy between the surface of the BN and AlN nanoribbons and nitrogen dioxide (NO_2_) gas molecules was computed via the following relation [27]:

(1) $$E_{Ads.}=\left(E_{Gas}+E_{Nanoribbon}\right)-E_{Complex}$$

where ($E_{Gas}$), ($E_{Nanoribbon}$) and ($E_{Complex}$) are the total energies for NO_2_ gas molecules, nanoribbons (BN and AlN) and the adsorbed system, respectively.

The energy gap ($E_{g}$) can be computed by the following relation:

(2) $$E_{g}=E_{LUMO}-E_{HOMO}$$

where ($E_{LUMO}$) and ($E_{HOMO}$) represent the lower unoccupied molecular orbital and higher molecular orbital energies, respectively.

The selectivity (*S*) can be estimate from the following expression [28]:

(3) $$S\left(\%\right)=Abs(\frac{E_{0}-E_{1}}{E_{0}})$$

where ($E_{0}$) and ($E_{1}$) represent the energy gap value in the absence and presence of NO_2_ gas molecules, respectively. In addition, we can modify Formula (3) to determine the optical response based on the change in the optical band gap as follows:

(4) $$Optical\;response\;\left(OR\right)=\frac{E_{\lambda_{0}}-E_{\lambda_{1}}}{E_{\lambda_{0}}}$$

where $E_{\lambda_{0}}$ and $E_{\lambda_{1}}$ refer to the optical band gap in the absence and presence of NO_2_ gas molecules, respectively.

## 3. Structural Properties of the BN and AlN Nanoribbons

**Figure 1:**
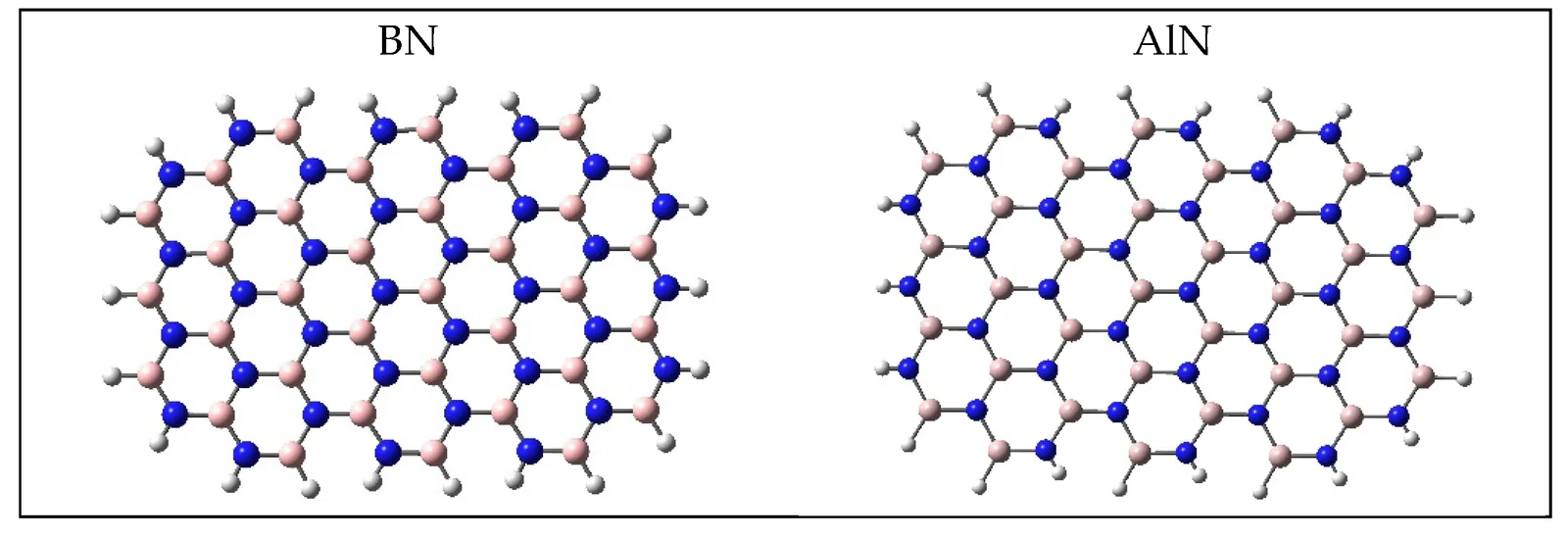
The relaxed geometrical structures of BN and AlN nanoribbons measured via DFT method.

In this section, the structural properties of BN and AlN nanoribbons are investigated. The structural characteristics involve the bond length and angle length between atoms. The ground-state characteristics were deduced by density functional theory (DFT). Figure 1 shows the geometrical relaxation structural of the BN and AlN nanoribbons. The BN and AlN nanoribbons have a (4,4) armchair configuration with a length of approximately 1.4 nm. Six different adsorption configurations were investigated, including three adsorption configurations for each nanoribbon. Additionally, the structural model contains a total of 90 atoms composed of boron, nitrogen, aluminum, and hydrogen atoms.

Notice that each nitrogen (N) atom formed three covalent bonds with three neighboring boron (B) atoms in the BN nanoribbon and three covalent bonds with aluminum (Al) atoms respectively. The covalent bond between B and N was observed around 1.425–1.460 Å and that between Al and N was observed around 1.816–1.822 Å in the BN and AlN nanoribbons, respectively. The calculated bond length between B-N and Al-N agrees with the finite hexagonal boron nitride and aluminum nitride nanoribbons in [29], showing that the finite nanoribbon model preserves the intrinsic structural properties of the periodic system. Table 1 shows the bond lengths and bond angles between the atoms in the BN and AlN nanoribbons. The DFT calculation for geometrical relaxation indicated that all geometrical parameters agreed with the previous reports in [30,31].

## 4. Adsorption Energy for NO_2_ Gas Molecules on the Surface of BN Nanoribbon

**Figure 2:**
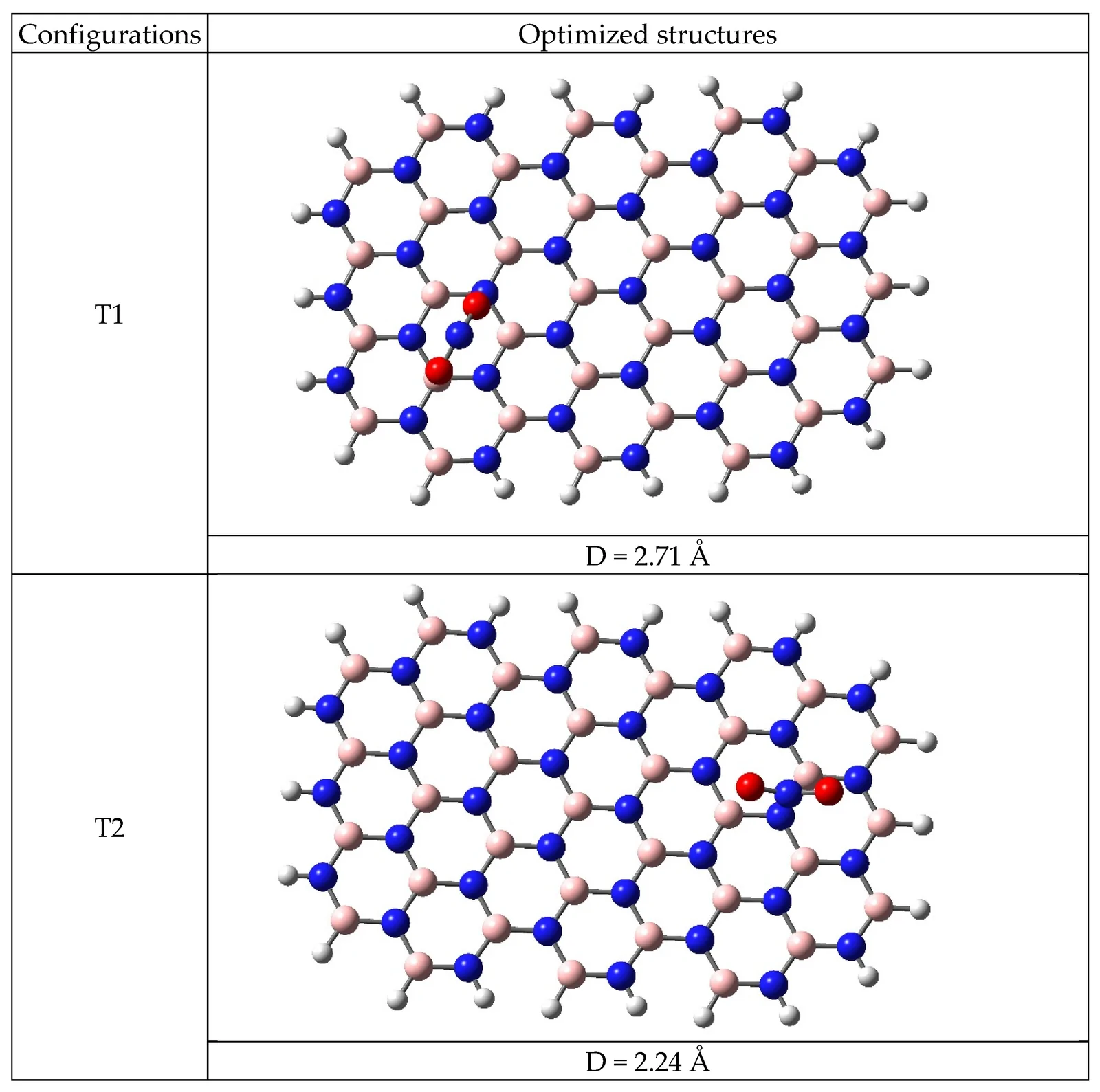

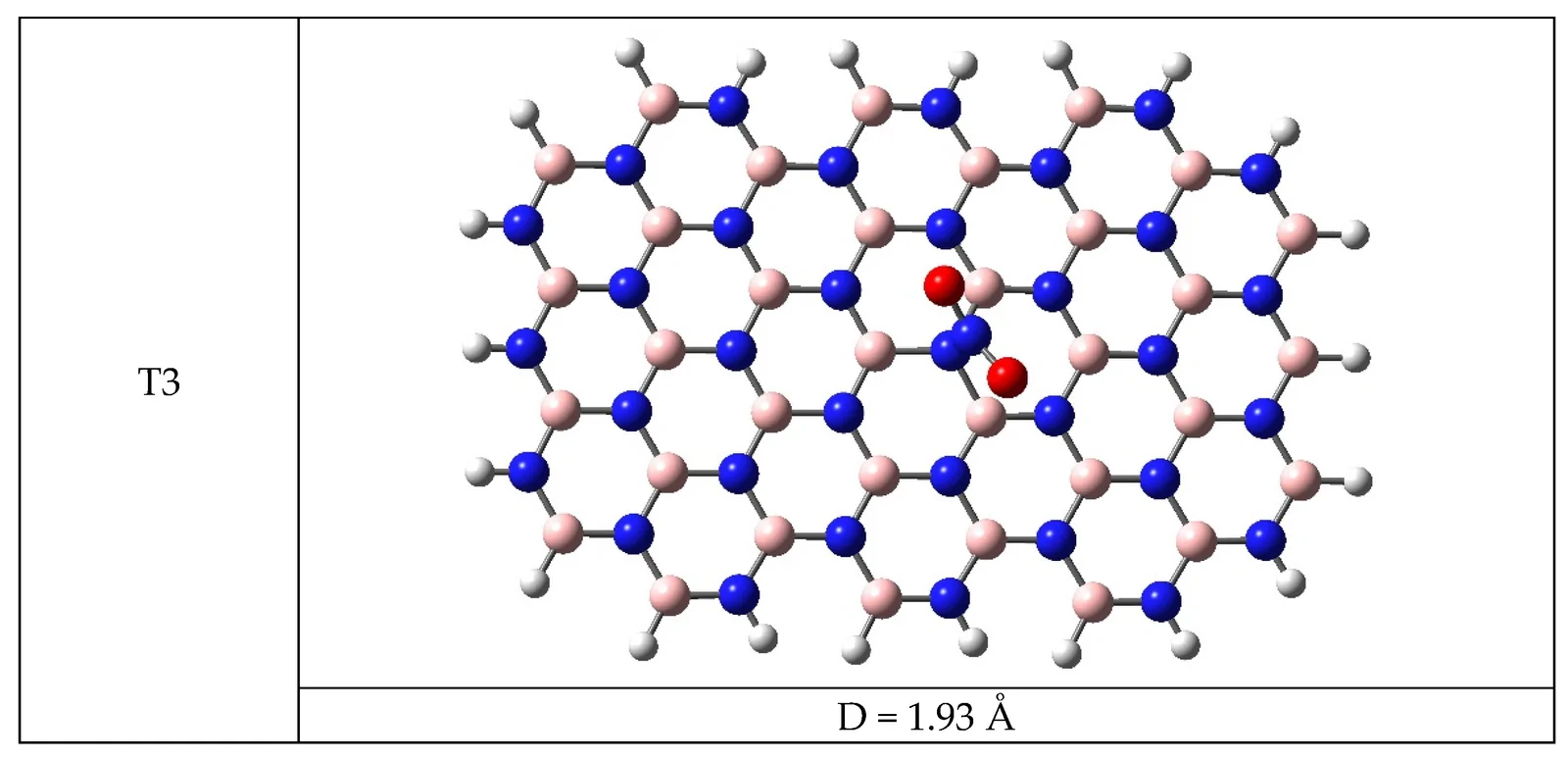
The most optimized geometrical structures for NO_2_ gas molecule adsorption configuration on the surface of BN nanoribbons. The blue, red, white, gray and shallow gray balls are nitrogen, oxygen, hydrogen, boron and aluminum, respectively.

In the present section, the adsorption behavior of NO_2_ gas molecules on the BN nanoribbon surface is evaluated via adsorption energy, adsorption distance, charge transfer molecular orbital analysis and sensing performance. Three possible configurations (T1, T2 and T3) were considered to investigate the interaction between NO_2_ gas molecules and the BN nanoribbon; Figure 2 shows the optimized adsorption configurations considered in the present study. The evaluated adsorption energies conclude a strong energy chemisorption between the NO_2_ gas molecule and the surface of the BN nanoribbon; the adsorption energy strength follows the order T3 > T2 > T1, showing that configuration T3 is the most energetic of the configurations. Additionally, the optimized adsorption distance was evaluated to be 2.71, 2.24 and 1.93 for T1, T2 and T3, respectively. Thus, configuration T3 shows the strongest adsorption because the NO_2_ molecule is localized closer to the BN surface, resulting in stronger orbital overlap and chemical interaction. Furthermore, the relaxed BN nanostructure conserves its planer surface geometry after NO_2_ adsorption, concluding that the adsorption mechanism does not induce substantial structural distortion.

Charge transfer (CT) calculation is an essential tool for determining both the magnitude and direction of charge transfer between gas molecules and the nanomaterial surface. The direction of transferred charge depends on the sign of the evaluated net charge. A positive value shows that electrons are transferred from the NO_2_ molecule to the BN nanoribbon, while a negative value shows electron transfer in the opposite direction [32]. The Mulliken charge analysis indicates that the transferred charge increases from +0.07 to +0.1, following the same trend as adsorption energy. These outcomes illustrate that stronger adsorption energy is accompanied by high charge transfer between the gas molecule and the BN nanoribbon surface. Moreover, the +ve charge value proves that electrons are transferred from the NO_2_ gas molecule to the BN nanoribbon surface; thus, NO_2_ acts like an electron donor during the adsorption interaction.

Overall, based on the adsorption energy, adsorption distance and charge transfer analysis, configuration T3 was recognized as the most stable adsorption site for NO_2_ on the BN nanoribbon. In summary, to understand the impact of adsorption energy on the sensing efficiency, the electronic characteristics of the optimized configurations are discussed in the following sections. Table 2 illustrates the adsorption energy, adsorption distance and charge transfer results of the NO_2_ configurations.

## 5. Adsorption Energy for NO_2_ Gas Molecules on the Surface of AlN Nanoribbon

**Figure 3:**
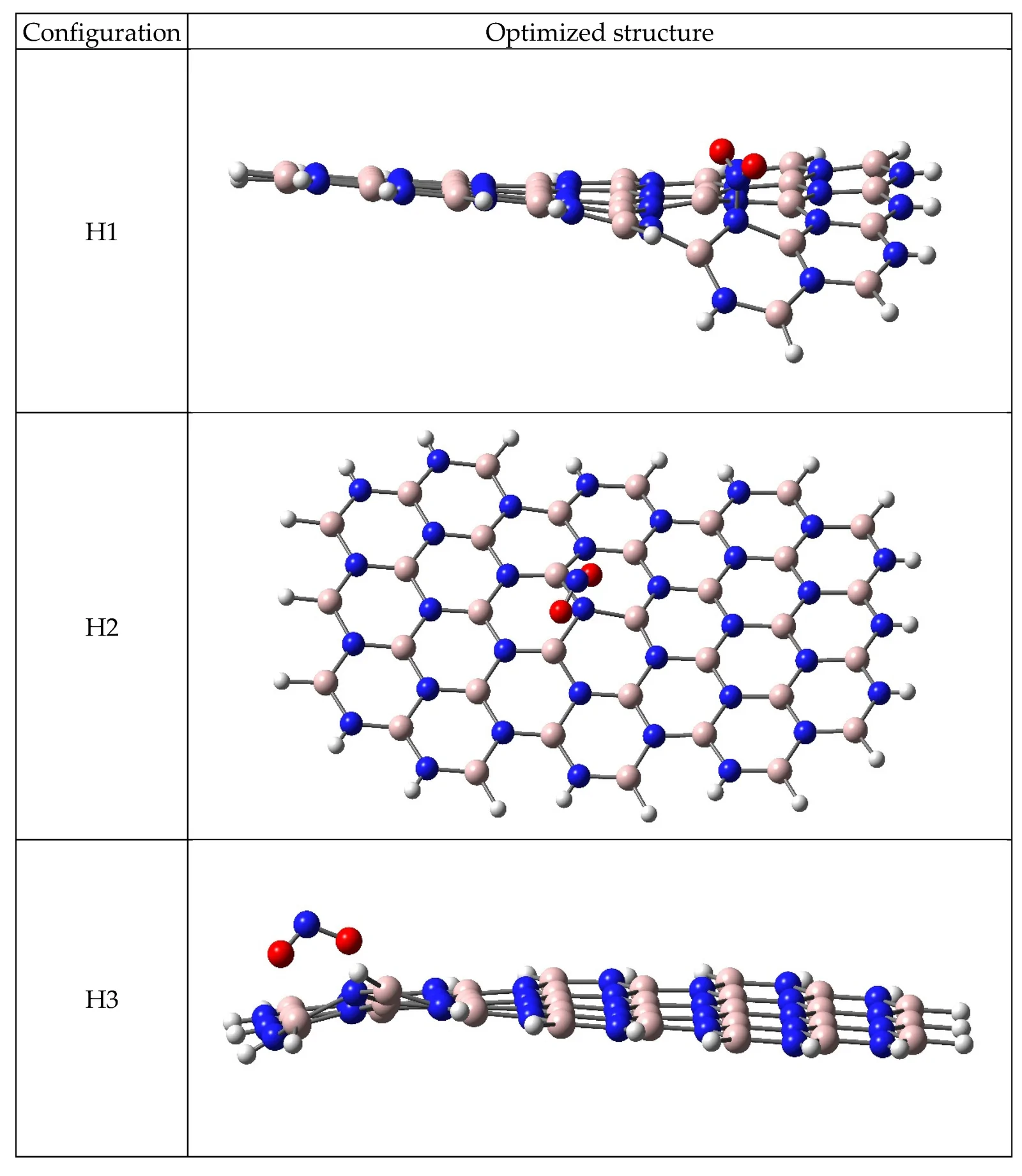
The most optimized geometrical structures for NO_2_ gas molecule adsorption configuration on the surface of AlN nanoribbons. The blue, red, white, gray and shallow gray balls are nitrogen, oxygen, hydrogen, boron and aluminum, respectively.

In the following section, the adsorption behavior of NO_2_ gas molecules on the AlN nanoribbon surface is evaluated. Similar to BN nanoribbons, three possible adsorption orientations (H1, H2 and H3) were considered to evaluate interaction energy between the NO_2_ molecule and AlN nanoribbon surface. The optimized adsorption geometries for the evaluated configurations are presented in Figure 3. The calculated adsorption energies indicate strong chemosorption between the NO_2_ gas molecule and AlN nanoribbon surface. The adsorption strength follows the order H1 > H2 > H3, showing that H1 is the most energetically favorable adsorption configuration. Due to the strong chemosorption interaction, observable local structural deformation was noticed on the AlN nanoribbon surface after NO_2_ adsorption. The observed structural defect modified the interaction strength between the NO_2_ gas molecule and the AlN nanoribbon, presenting a strong adsorption behavior. The optimized adsorption distance for the H1 configuration is approximately 1.43, which is the shortest among all studied configurations. Therefore, the H1 configuration is noted as the most favorable adsorption site due to having the shortest adsorption optimization distance. Overall, the evaluated adsorption energies range from −4.2 eV to −5 eV, indicating that NO_2_ is strangely chemisorbed on the AlN nanoribbon surface. Furthermore, the structural deformation observed after adsorption is consistent with shorted optimized adsorption distance and relatively high adsorption energies, indicating a strong chemical interaction between NO_2_ molecules and the AlN nanoribbon surface.

Table 3 shows the computed adsorption energy, adsorption distance and charge transfer for AlN nanoribbons. The Mulliken charge transfer analysis revealed that the net charge transferred between NO_2_ gas molecules and the AlN nanoribbon surface ranges from −0.111 to −0.161 e. Since the net charge is negative, electrons are transferred from the AlN nanoribbon surface to NO_2_ molecules. Thus, the NO_2_ gas molecules act as electron acceptors during the adsorption process. Moreover, the H1 configuration exhibits the largest charge transfer, which is consistent with its highest adsorption energy and shortest adsorption distance. In addition, the structural deformation modifies the electronic interaction between the NO_2_ molecule and the AlN nanoribbon, leading to greater charge transfer. Overall, the adsorption energy and charge transfer results conclude that the AlN nanoribbon exhibits stronger chemosorption toward NO_2_ than BN. This modified interaction is ascribed to the local structural deformation induced after adsorption.

## 6. Electronic Properties and Sensitivity of BN/AlN Nanoribbons for NO_2_ Adsorption

In the following section, the effect of NO_2_ gas adsorption on the electronic properties of BN and AlN nanoribbons is evaluated. The electronic parameters, including HOMO, LUMO and Eg, are computed to understand the sensing process of the proposed nanostructures. Changes in the frontier molecular orbital energies after gas adsorption provide valuable information regarding the electronic interaction between NO_2_ molecules and the nanostructure surface. Moreover, energy gap is considered one of the most essential parameters controlling gas-sensing efficiency, since even a slight variation in its value can significantly influence the electrical conductivity and sensing response of the materials. Therefore, the energy gap is adopted as the primary parameter for computing the sensitivity of the studied nanostructures toward NO_2_ gas molecules. Table 4 lists the molecular orbital energies, energy gap and sensitivity of BN and AlN nanostructures after adsorption.

The evaluated electronic properties show that strong chemosorption has a significant influence on the molecular orbital energy and energy gap of the BN nanoribbon. After NO_2_ adsorption, both the HOMO and LUMO energy levels shift toward lower energy values, indicating a strong electronic interaction between the gas molecule and the nanoribbon surface.

For instance, the LUMO energy level decreases from −1.444 eV to −7.491 eV after NO_2_ adsorption. Furthermore, the energy gap decreases remarkably from approximately 6.2 eV to 0.401 eV, showing that the electron excitation between HOMO and LUMO becomes much easier after adsorption. Since the pure BN nanoribbon is an insulating material with a wide band gap, this remarkable reduction in the energy gap is expected to enhance its electrical conductivity and gas-sensing efficiency.

The evaluated sensitivity ranges from 81% to 93%, showing the excellent sensing capability of the BN nanoribbon toward NO_2_ molecules.

For the AlN/NO_2_ configurations, the evaluated molecular orbital energy levels shift towards lower energy values, following the same trend observed for the BN/NO_2_ systems. The pure AlN nanoribbon is a wide-band-gap semiconductor with an energy gap of approximately 4.8 eV. Owing to strong chemosorption between NO_2_ gas molecules and the AlN nanoribbon surface, the band gap decreases dramatically to 0.130 eV for the most favorable configuration. Therefore, electron excitation from HOMO to LUMO needs considerably lower energy, resulting in an enhanced electronic response of the AlN nanoribbon. The computed sensitivity results showed that the AlN nanoribbon exhibits superior sensing performance towards NO_2_ gas molecules than the BN nanoribbon. The maximum sensitivity regarding the H3 configuration reaches approximately 97%, showing the excellent sensing ability of the AlN nanoribbon.

Overall, both BN and AlN nanoribbons show notable improvements in their electronic properties after NO_2_ adsorption, thus enhancing sensing efficiency. However, the limitation of the electronic response difference depends on the adsorption configuration.

Interestingly, although the T1 and H1 configurations exhibit the strongest chemisorption, the T2 and H3 configurations demonstrate the highest sensitivity. This observation shows that adsorption energy primarily reflects the interaction strength between gas molecules and the nanoribbon surface, while the sensing performance is mainly controlled via the electronic structure modification, especially the reduction in the energy gap.

**Figure 4:**
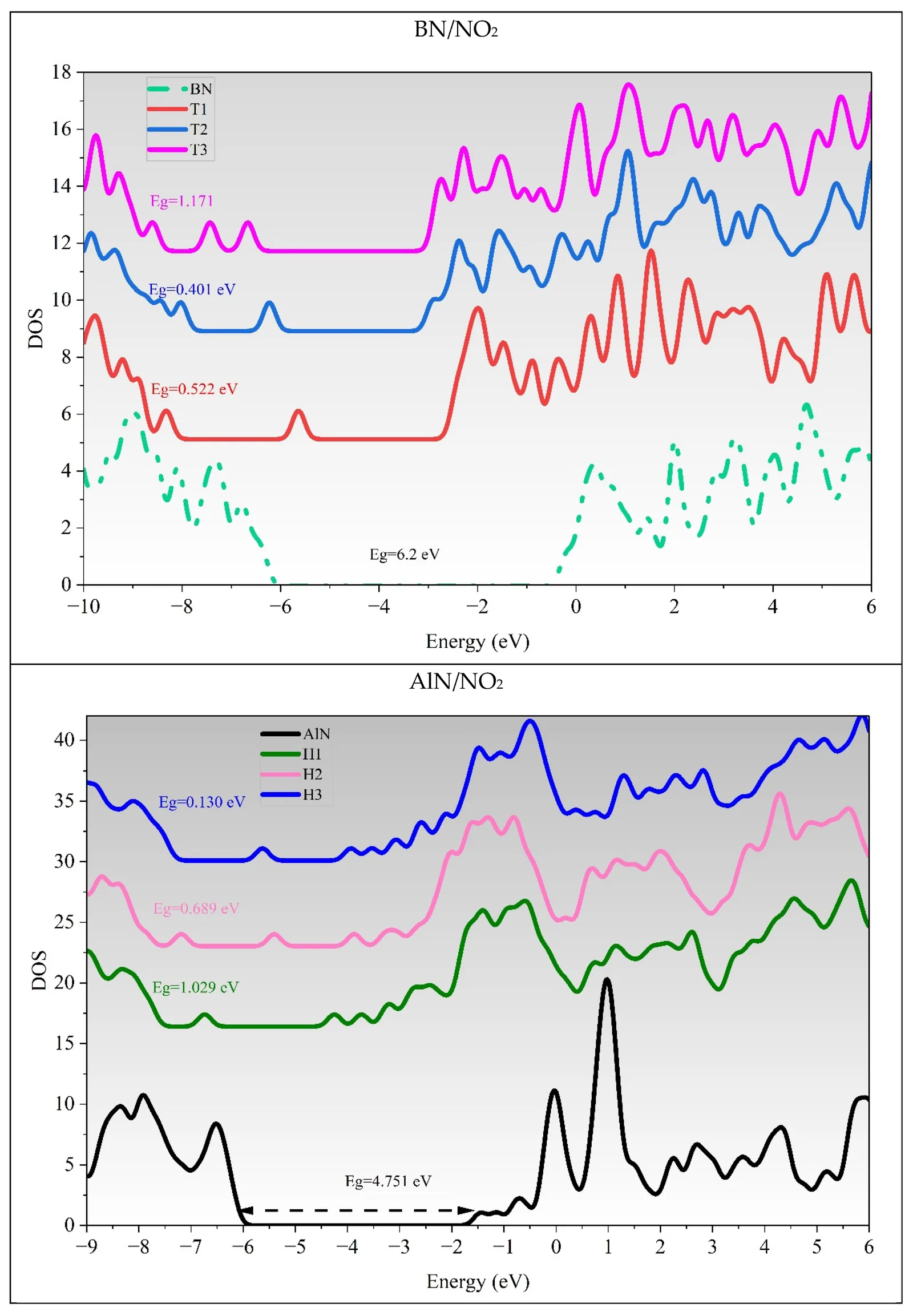
Total density of states (DOS) spectra of pristine BN and AlN nanoribbons, together with their NO_2_ adsorption configurations (T1–T3 for BN and H1–H3 for AlN). The green and black dashed line represents the energy gap of the pure BN and AlN nanoribbons.

Overall, Figure 4 represents the density of states (DOS) spectra of the pure BN and AlN nanoribbons and those with NO_2_ adsorbed on their surfaces. As is shown, NO_2_ significantly improves the electronic structures of both nanoribbons. For BN/NO_2_ configurations, the band gap becomes narrower, accompanied by an observable shift of the molecular orbitals toward lower energy ranges. A similar trend is recognized regarding AlN/NO_2_ configurations, where NO_2_ adsorption induces a remarkable reduction in the band gap energy, and the molecular energy levels shift toward lower energy values. These results are in good agreement with HOMO/LUMO analysis, showing that NO_2_ interaction effectively modifies the electronic response of both BN and AlN nanoribbons.

## 7. Molecular Orbital Charge Distribution

**Figure 5:**
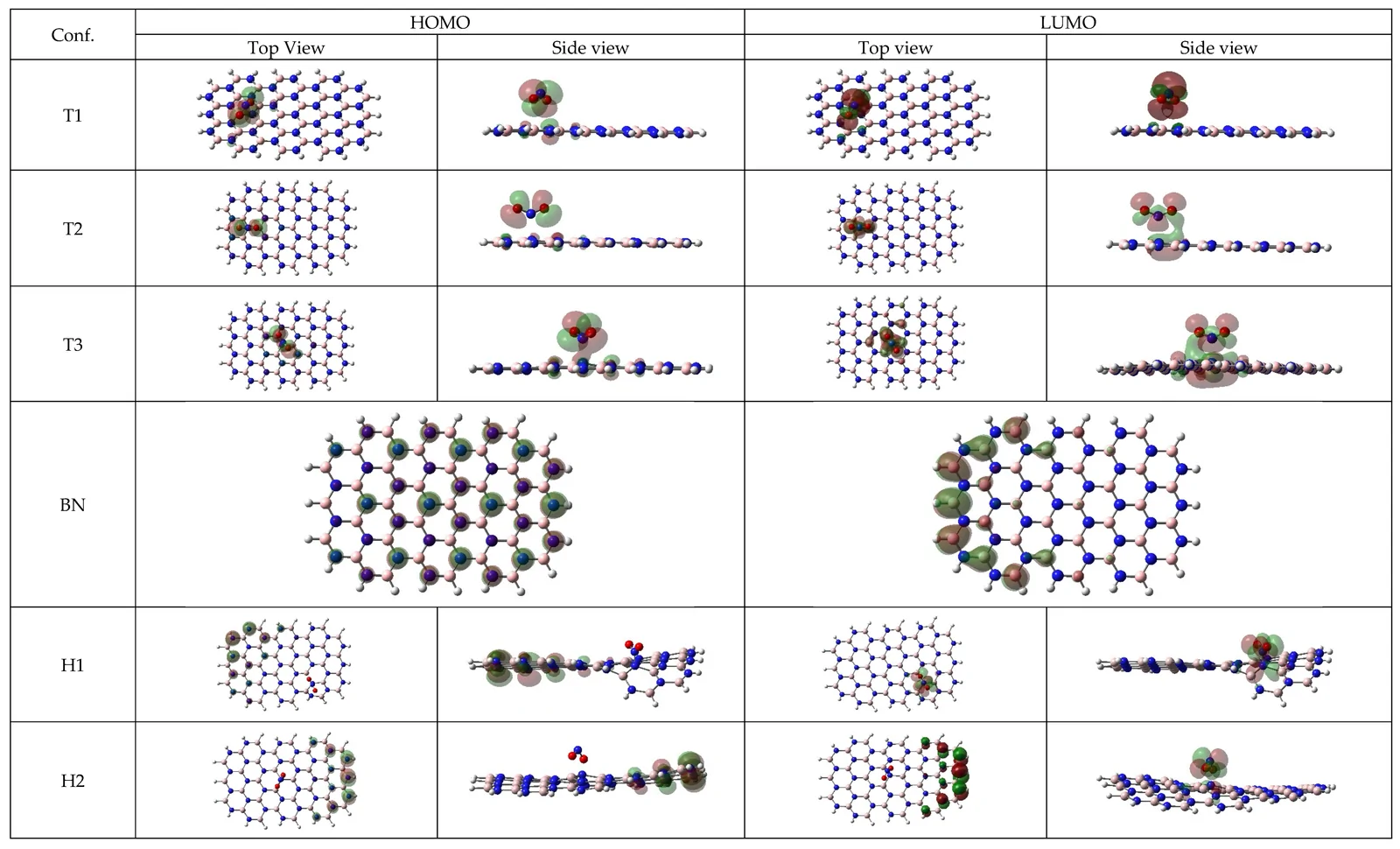

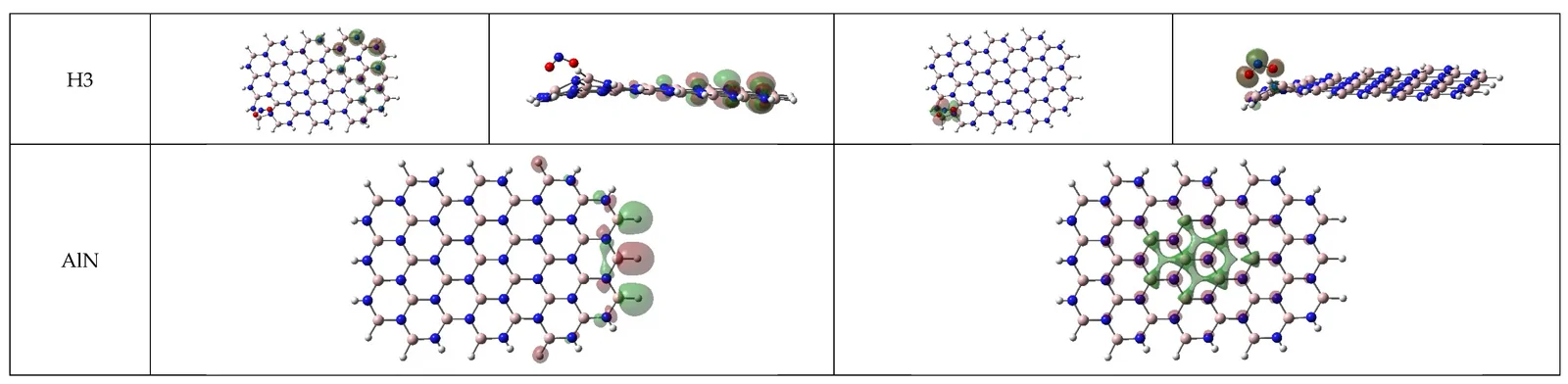
The electron charge density around the surface of BN and AlN nanoribbons before and after NO_2_ gas molecule adsorption. The green and red spheres represent the positive and negative values of the electron charge density, respectively.

The frontier molecular orbital (FMOs) distributions were analyzed to study the charge redistribution induced via NO_2_ adsorption on BN and AlN nanoribbons. Figure 5 shows the HOMO and LUMO distributions for the three adsorption configurations of the BN and AlN nanoribbons. For the pure BN nanoribbon, the HOMO is mainly localized on the nitrogen atoms of the nanoribbon; in contrast, the LUMO is distributed along the ribbon edge. After NO_2_ adsorption on the BN nanoribbon surface, a remarkable redistribution of the FMOs is observed. In the T1 and T2 configurations, the HOMO extends over both the adsorption site and the NO_2_ molecule, showing orbital overlap between the adsorbate and the BN surface. Meanwhile, the LUMO becomes extremally localized around the NO_2_ gas molecule and neighboring adsorption site, suggesting efficient electron acceptance by the adsorbed NO_2_ molecule.

A similar trend is observed for the AlN nanoribbon. The HOMO and LUMO are mainly distributed over the nanoribbon surface. Following NO_2_ adsorption, the HOMO shifts toward the adsorption region, while the LUMO becomes highly localized on the NO_2_ molecule and adjacent surface atoms. The redistribution shows strong electron coupling between the adsorbate and the AlN nanoribbon and is consistent with the calculated charge transfer and adsorption energy results.

Overall, the recognized redistribution of the HOMO and LUMO around the adsorption site indicates the strong chemosorption of NO_2_ gas molecules on both BN and AlN nanoribbons. The significant orbital overlap enhances charge transfer across the interface, improving the electronic structure, which is directly responsible for the modified sensing efficiency of the proposed nanoribbons. 

## 8. UV–Visible Spectrum and Optical Response (OR)

In the present section, the effect of NO_2_ adsorption on the optical properties of the BN and AlN nanoribbons is examined via time-dependent density functional theory (TD-DFT). The changes in the UV–Visible absorption spectra and optical band gap provide essential insights into the optical response of the proposed nanoribbon towards NO_2_ gas molecules [33]. The enhanced optical response after gas molecule adsorption shows the potential of BN and AlN nanoribbons for the development of optical gas-sensing devices. Therefore, the maximum wavelength of absorption, optical band gap and optical response of the BN/NO_2_ and AlN/NO_2_ adsorption configurations are analyzed and correlated with adsorption strength. Table 5 summarizes the evaluated maximum absorption wavelengths of absorption, optical band gap and optical response parameters of the different adsorption configurations.

TD-DFT calculations show that the maximum absorption wavelength of pristine BN is located at 200.96 nm, corresponding to an optical band gap of 6.20 eV. The evaluated value of λmax is in agreement with experimental and theoretical studies in [28]. After NO_2_ adsorption, the absorption spectra show a pronounced red shift toward longer wavelengths, showing a significant reduction in the excitation energy. Among all adsorption configurations, the T1 configuration illustrates the largest red shift and smallest optical excitation energy. This behavior is due to the strong improvement of the electronic structure induced via NO_2_ interaction, which modifies the optical transition probability. Furthermore, the evaluated OR ranges from approximately 67.7% to 71.0%, with the T1 system illustrating the strongest optical response of these configurations.

For the pristine AlN nanoribbon, the maximum absorption wavelength is observed at approximately 270 nm, corresponding to an optical energy gap of 4.59 eV. After NO_2_ adsorption, the UV–Visible absorption spectra exhibit a pronounced red shift towards longer wavelengths, indicating a notable reduction in the optical excitation energy. The optical behavior originates from the improvement in electronic structures induced by the interaction between the NO_2_ molecules and the AlN nanoribbon surface. Moreover, the optical response improves remarkably from 14.2% to 48.1% after gas adsorption. In summary, the T3 gas adsorption configuration exhibits the best optical response among other configurations, demonstrating the superior optical sensing capability of AlN towards NO_2_ molecules.

**Figure 6:**
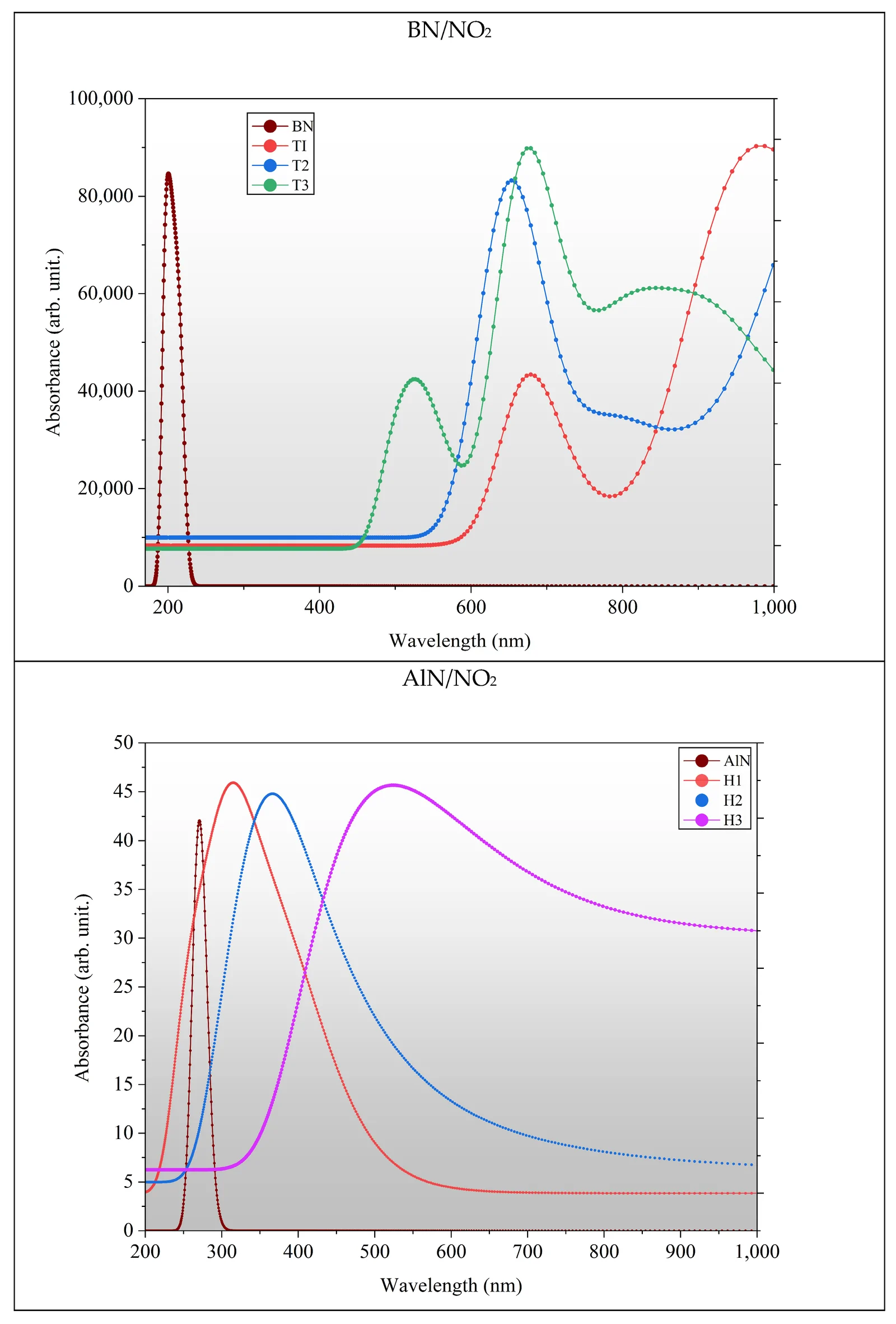
The evaluated UV–Visible spectra of the BN/NO_2_ and AlN/NO_2_ adsorption configurations.

Overall, the evaluated results show that the BN/NO_2_ adsorption configurations exhibit greater optical responses than the corresponding AlN/NO_2_ systems. The expressive variation in the optical band gap after NO_2_ adsorption confirms the excellent potential of BN nanoribbons for optical gas-sensing applications. Although the AlN nanoribbons also presetn a notable optical response, their performance is comparatively lower than the BN nanoribbons. Figure 6 shows the evaluated UV–Visible spectra of the BN/NO_2_ and AlN/NO_2_ adsorption configurations.

## 9. Thermochemistry Properties and Response Time

Based on the adsorption energy, sensitivity and optical response calculations, the NO_2_ gas molecule shows remarkable chemisorption on the surface of BN and AlN nanoribbons. In addition, the BN nanoribbon exhibits a strong optical response, while the AlN nanoribbon illustrates stronger adsorption behavior. In the present section, the thermodynamic parameters of Gibbs free energy (ΔG) and enthalpy (ΔH) are computed to determine whether the adsorption process between the BN and AlN nanoribbons is spontaneous or non-spontaneous and whether the interaction is exothermic or endothermic, respectively. A higher negative value of Gibbs free energy shows that the adsorption process is spontaneous, while a positive value corresponds to a non-spontaneous process. In addition, a more negative value of (ΔG) implies a more thermodynamically favorable process. In the same vein, negative values of ΔH correspond to an exothermic adsorption process, while positive values indicate an endothermic interaction. The Gibbs free energy and enthalpy can be calculated by the following equestions:

(5) $$∆G={∆G}_{Product}-{∆G}_{Reactans\;}\;$$

(6) $$∆H={∆H}_{Product}-{∆H}_{Reactans\;}$$

Table 6 illustrates the evaluated Gibbs free energy and enthalpy for the different NO_2_ adsorption configurations on the BN and AlN nanoribbon surfaces measured in eV units. According to a previous study in Ref [34], an adsorption system with |G| > 0.4 eV can be classified as chemosorption, while values less than 0.4 eV correspond to physisorption. As shown in Table 6, all evaluated Gibbs free energy values are significantly larger than 0.4 eV in absolute terms and remain negative, concluding that the adsorption of NO_2_ on both BN and AlN nanoribbons is spontaneous and belongs to the chemosorption regime. Consequently, all evaluated enthalpy values are negative, showing that the adsorption process is exothermic. Computed thermodynamic parameters follow the same trend as the adsorption energy and enthalpy values, where the configuration with stronger adsorption shows more negative Gibbs free energy and enthalpy results. For instance, the T3 configuration of BN and H1 configuration of AlN possess the most negative Gibbs free energy and enthalpy, confirming their high thermodynamic stability among the evaluated adsorption configurations.

Finally, the recovery behavior is considered an essential factor for computing the practical performance of gas-sensing materials. The recovery time factor mainly depends on the desorption activation barrier, which describes the amount of energy required to remove the adsorbed gas molecule from the nanoribbon surface. According to the Arrhenius formula, the recovery time increases exponentially with increasing desorption activation energy, while it decreases with increasing temperature. Thus, strongly adsorbed gas molecules requires higher energy for desorption, resulting in a longer recovery time, while weakly adsorbed gas molecules are expected to show a faster recovery time trend.

In the present study, the adsorption energy, Gibbs free energy and enthalpy calculations show that the interaction between the NO_2_ gas molecule and the AlN nanoribbon is stronger than for BN. AlN adsorption configurations show more negative adsorption energies and Gibbs free energies, implying higher thermodynamic stability of the adsorbed complexes. From a physical point of view, these results indicate that removing the NO_2_ molecule from the AlN nanoribbon surface requires higher energy than for the BN nanoribbon surface. Thus, the BN adsorption configurations are expected to show faster recovery time behavior than AlN systems.

Overall, the above investigation is based on the physical analysis of the adsorption strength and thermodynamic properties. A quantitative evaluation of the recovery time requires the determination of the desorption activation barrier using transition state calculations. Because transition state calculations were not performed in the present work, the recovery time is explained qualitatively rather than quantitatively.

## 10. FT-IR Spectroscopy

**Figure 7:**
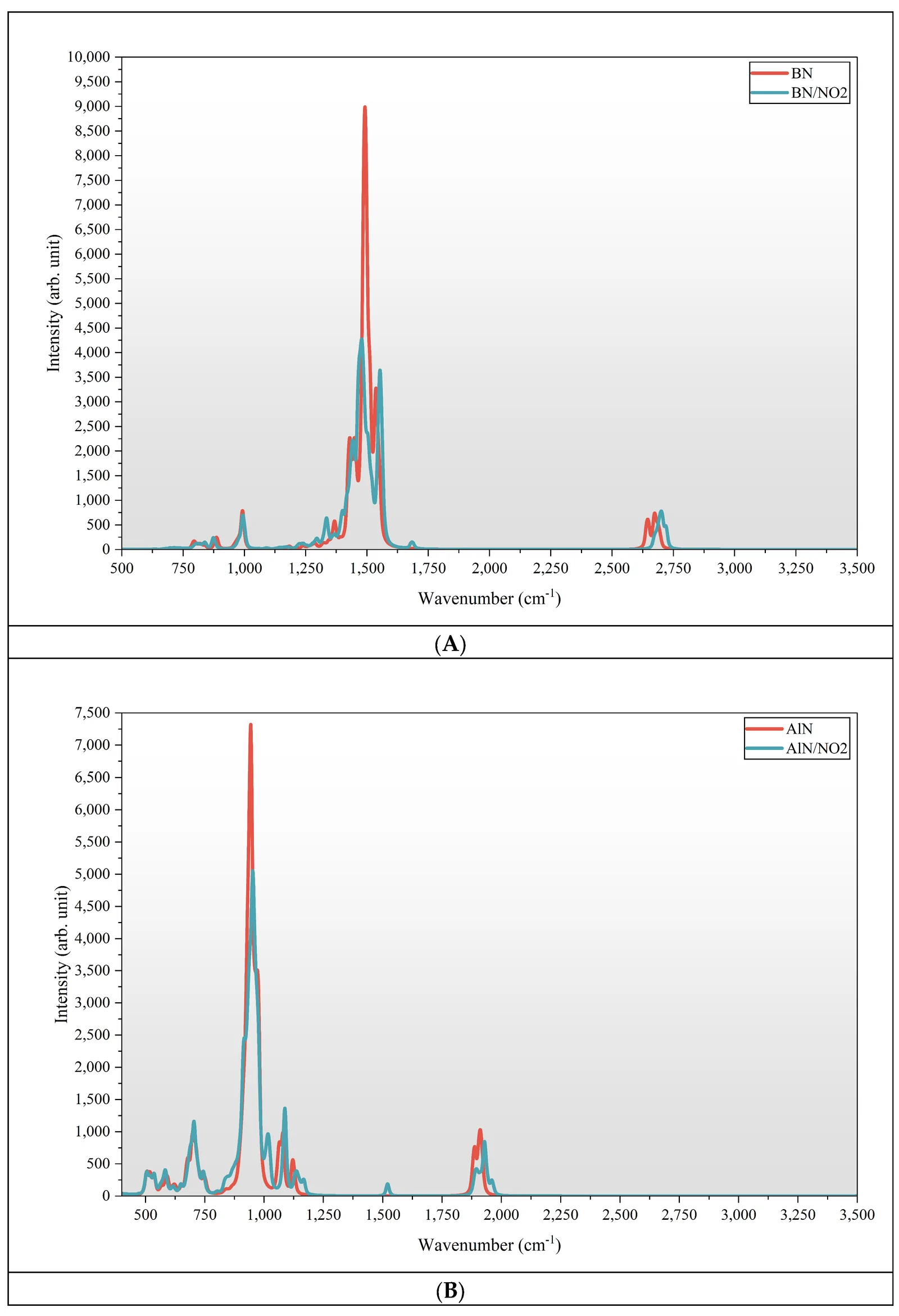
(**A**) The vibration spectrum regarding BN nanoribbon in the pure and presence of the NO_2_ gas molecule (T1 configuration). (**B**) Illustrates the vibration spectrum regarding AlN nanoribbon in the pure and presence of the NO_2_ gas molecule (H3 configuration).

In the present section, the structural properties of BN and AlN nanoribbons are examined by FT-IR spectrum calculation. The FT-IR spectrum is examined in the absence and presence of NO_2_ gas molecules; after the interaction, we evaluate the possibility of determining the N-O vibration band. Figure 7A shows the vibration spectrum of the BN nanoribbon. For the pure BN nanoribbon, the fundamental B-N vibration bands are observed at the wavenumbers 1465.65 cm^−1^ and 1501.45 cm^−1^, for the bending and stretching modes, respectively. In the same vein, the two secondary absorption bands of B-H and N-H, corresponding to stretching mode, are found at the peaks of 3594.53 cm^−1^ and 2691.84 cm^−1^, respectively. In addition, the bending mode for these groups is localized at the peaks 872.42 cm^−1^ and 791.41 cm^−1^. The present results are in agreement with experimental and theoretical studies in [35]. During the interaction of the NO_2_ gas molecule on the surface of the BN nanoribbon, new vibration bands occur in the presence of NO_2_ gas. An asymmetric stretching band appears with a low-intensity peak at 1540.55 cm^−1^. Furthermore, the symmetric stretching vibration mode peak is found at 1344.55 cm^−1^. In addition, the results show that there is a small shift in the longer frequency range, especially in the B-H absorption peak.

Additionally, the FT-IR spectrum of the AlN nanoribbon is examined. Figure 7B shows the FT-IR spectrum for AlN in the absence and presence of NO_2_ gas molecules. The fundamental Al-N vibration peak is observed at the wavenumber value of about 688.22 cm^−1^ (bending mode). In addition, the stretching vibration peak appears at 910.22 cm^−1^. The present theoretical calculations approximately agree with the experimental study in [34]. During the adsorption process, the FT-IR spectrum shows that a new vibration peak appears at 1525.22 cm^−1^, belonging to the N-O absorption band in asymmetric stretching mode. Similar to the BN/NO_2_ adsorption process, the chemical adsorption between the gas molecule and surface of the AlN nanoribbon causes a shift in the vibration spectrum [35].

## 11. Conclusions

DFT, DFT-D3 and TD-DFT calculations were utilized to evaluate the adsorption behavior of NO_2_ gas molecules on the surface of BN and AlN nanoribbons, in addition to their electronic and optical response characterizations. The adsorption calculation confirmed stronger chemosorption on the surface of the AlN nanoribbon than the BN nanoribbon. The sensitivity results illustrated that the BN nanoribbon exhibits higher sensitivity performance among the adsorption configurations, while the highest electronic sensitivity was determined for the H3 adsorption configuration of the AlN nanoribbon surface. Consequently, the BN nanoribbon exhibits a superior optical response to the AlN nanoribbon. Overall, the results are promising: BN nanoribbons are candidates for applications in optical gas-sensing devices, while AlN possesses high performance for electronic NO_2_ gas-sensing devices.

## Figures and Tables

**Table 1:** The bond length and angle length between the atoms in the BN and AlN nanoribbons measured in angstroms (Å) and degrees (deg.), respectively.

Nanoribbons	Bond Types	Bond Length	Angle Types	Bond Angle
BN	B−N	1.425–1.460	B−N−B	119.687–122.680
	B−H	1.190–1.193	N−B−N	119.876–122.414
	N−H	1.010–1.012	B−N−H	117.942–119.241
			N−B−H	118.332–120.800
AlN	Al−N	1.816–1.823	Al−N−Al	118.617–123.412
	Al−H	1.596–1.600	N−Al−N	118.199–123.580
	N−N	1.016–1.017	N−Al−H	120.266–122.131
			Al−N−H	117.459–118.805

**Table 2:** Evaluated adsorption parameters, including adsorption energy (Ead), adsorption distance (D (Å)) and Mulliken charge transfer (CT), regarding optimized NO_2_ adsorption configurations on the BN nanoribbon surface.

Configuration	Ead (eV)	D (Å)	CT e
T1	−2.768	2.71	0.046
T2	−2.888	2.24	0.077
T3	−3.062	1.93	0.10

**Table 3:** Evaluated adsorption parameters, including adsorption energy (Ead), adsorption distance (D (Å)) and Mulliken charge transfer (CT), of optimized NO_2_ adsorption configurations on the AlN nanoribbon surface.

Configuration	Ead (eV)	D (Å)	CT e
H1	−5.015	1.43	−0.161
H2	−4.471	2.54	−0.159
H3	−4.225	2.94	−0.111

**Table 4:** The the evaluated frontier molecular orbital energy, band gap energy and sensitivity regarding BN and AlN nanoribbons with respect to NO_2_ different adsorption configurations.

BN/NO_2_ Adsorption Configurations				
Configuration	HOMO (eV)	LUMO (eV)	Eg (eV)	S%
T1	−8.8482	−8.3252	0.522	91.52
T2	−8.4267	−8.0258	0.401	93.50
T3	−8.6016	−7.4302	1.171	81.02
**BN**	**−6.3665**	**−0.1921**	**6.20**	**----**
**AlN/NO_2_ Adsorption configurations**				
Configuration	HOMO (eV)	LUMO (eV)	Eg (eV)	S%
H1	−7.7673	−6.7377	1.029	78.33
H2	−7.8775	−7.1881	0.689	85.48
H3	−7.6212	−7.4907	0.130	97.25
**AlN**	**−6.1962**	**−1.444** **3**	**4.751**	**----**

**Table 5:** The maximum wavelength of absorption, optical band gap and optical response parameters for BN/NO_2_ and AlN/NO_2_ adsorption configurations.

BN/NO_2_ Adsorption Configurations			
Configuration	λ(max) (nm)	E(opt) (eV)	OR (%)
**BN**	200	6.20	--
T1	670	1.85	70.9 (~71.0)
T2	690	1.80	67.7
T3	620	2.00	70.2
**AlN/NO_2_ Adsorption configurations**			
Configuration	λ(max) (nm)	E(opt) (eV)	OR (%)
**AlN**	270	4.59	--
H1	315	3.94	14.2
H2	365	3.40	25.9
H3	520	2.38	48.1

**Table 6:** The Gibbs free energy, enthalpy, and types of adsorption for NO_2_ gas molecule adsorption configurations on BN and AlN nanoribbon surfaces.

BN/NO_2_ Adsorption Configurations			
Configuration	∆G (eV)	∆H (eV)	Types of adsorptions
T1	−2.484	−2.823	Chemisorption
T2	−2.563	−2.905	Chemisorption
T3	−2.639	−3.0416	Chemisorption
**AlN/NO_2_ Adsorption Configurations**			
Configuration	∆G (eV)	∆H (eV)	Types of adsorptions
H1	−4.340	−4.821	Chemisorption
H2	−3.904	−4.331	Chemisorption
H3	−3.632	−4.113	Chemisorption

## Data Availability

The data presented in this study are available on request from the corresponding author. (These data are going to be part of a Ph.D. thesis, in preparation and not yet public. Due to University privacy policy, the data can be obtained from the corresponding author upon reasonable request.)
